# The physiological response of the deep-sea coral *Solenosmilia variabilis* to ocean acidification

**DOI:** 10.7717/peerj.5236

**Published:** 2018-07-20

**Authors:** Malindi J. Gammon, Dianne M. Tracey, Peter M. Marriott, Vonda J. Cummings, Simon K. Davy

**Affiliations:** 1 School of Biological Sciences, Victoria University of Wellington, Wellington, New Zealand; 2 National Institute of Water & Atmospheric Research, Wellington, New Zealand

**Keywords:** Deep-sea, Ocean acidification, Physiology, Scleractinian corals, Stony corals, Global change, Deep-sea corals

## Abstract

Several forms of calcifying scleractinian corals provide important habitat complexity in the deep-sea and are consistently associated with a high biodiversity of fish and other invertebrates. How these corals may respond to the future predicted environmental conditions of ocean acidification is poorly understood, but any detrimental effects on these marine calcifiers will have wider impacts on the ecosystem. Colonies of *Solenosmilia variabilis*, a protected deep-sea coral commonly occurring throughout the New Zealand region, were collected during a cruise in March 2014 from the Louisville Seamount Chain. Over a 12-month period, samples were maintained in temperature controlled (∼3.5 °C) continuous flow-through tanks at a seawater pH that reflects the region’s current conditions (7.88) and an end-of-century scenario (7.65). Impacts on coral growth and the intensity of colour saturation (as a proxy for the coenenchyme tissue that covers the coral exoskeleton and links the coral polyps) were measured bimonthly. In addition, respiration rate was measured after a mid-term (six months) and long-term (12 months) exposure period. Growth rates were highly variable, ranging from 0.53 to 3.068 mm year^−1^ and showed no detectable difference between the treatment and control colonies. Respiration rates also varied independently of pH and ranged from 0.065 to 1.756 µmol O_2_ g protein^−1^ h^−1^. A significant change in colour was observed in the treatment group over time, indicating a loss of coenenchyme. This loss was greatest after 10 months at 5.28% and could indicate a reallocation of energy with physiological processes (e.g.  growth and respiration) being maintained at the expense of coenenchyme production. This research illustrates important first steps to assessing and understanding the sensitivity of deep-sea corals to ocean acidification.

## Introduction

Deep-sea corals (Phylum Cnidaria) are an abundant and diverse group that are found worldwide and, like their shallow water counterparts, several groups are characterised by their ability to form calcium carbonate skeletons. Corals are vulnerable to environmental change resulting from anthropogenic disturbances such as climate change, ocean acidification (OA) (Guinotte et al., 2006; Turley, Roberts & Guinotte, 2007) and fishing (Clark & Rowden, 2009; Clark et al., 2015b). To date, no research has been carried out in the New Zealand region on the impacts of climate change, including OA, on this important group of scleractinian stony corals. One study by Thresher et al. (2011) in nearby Australian waters investigated the effects of chronic low carbonate saturation levels on the distribution, growth and skeletal chemistry of several deep-sea corals off southeastern Tasmanian seamounts and found that the distribution of scleractinian corals is constrained by low carbonate saturation levels.

Deep-sea corals are generally found in water temperatures between 4 and 12 °C (Roberts, Wheeler & Freiwald, 2006; Buhl-Mortensen & Mortensen, 2005). This largely corresponds to relatively shallow depths (between 50 and 100 m) at high latitudes, and greater depths (up to 4,000 m) at low latitudes (Roberts, Wheeler & Freiwald, 2006). However, solitary cup corals (e.g., *Caryophyllia antarctica, Gardineria antarctica* and *Flabellum impensum*) can be found up to 1,000 m deep in the high latitude waters of Antarctica (Parker & Bowden, 2010). Compared to the large numbers of shallow-water, reef building corals that have been described, only 10 deep-sea scleractinian reef-building species have been described globally (Cairns, 1979; Freiwald et al., 2004).

The South Pacific region, including New Zealand, supports a broad diversity of various deep-sea coral fauna (Williams et al., 2010), the majority of which live between depths of 200 and 1,200 m (Tracey et al., 2011). The scleractinain corals in the region are often found on elevated hard substrate with topographic complexity such as seamounts, knolls, on slope margins, ridges and canyons (Tracey et al., 2011), where they are in an advantageous position to feed in high current areas on particulate organic matter (Wolankski & Hamner, 1998). Branching forms of the scleractinian corals create three-dimensional reef structures in the deep, and these provide key biogenic habitat and refuge for many deep-sea invertebrates, fish and sharks. The community composition of various invertebrates associated with coral-reefs is well described in the literature (e.g., see Henry, Davies & Roberts, 2010). Fish have been seen on, or in close proximity to, stony and other habitat-forming deep-sea corals (Bongiorni et al., 2010; Soffker, Sloman & Hall-Spencer, 2011; Fossået al., 2012; Purser et al., 2013; Biber et al., 2013; Milligan et al., 2016), and the benefits of deep-sea reef habitats to shark species have also been reported (Henry et al., 2013).

Atmospheric concentrations of carbon dioxide (CO_2_) have increased since pre-industrial times due to anthropogenic emissions. The ocean acts as a carbon sink, absorbing this CO_2_, but results in changes to the chemistry of seawater, including a reduction in pH and the availability of free carbonate ions (Hoegh-Guldberg et al., 2007). By the end of this century, OA is expected to cause a decline in oceanic pH by 0.2–0.3 pH units (IPCC, 2013). This is in addition to the pH drop of 0.1 units, which has already been observed since pre-industrial times (Friedrich et al., 2012). OA is enhanced at low temperatures and high pressure, conditions experienced in the deep-sea (Orr et al., 2005; Roberts, Wheeler & Freiwald, 2006; Law et al., 2018). Already impacted by trawling (Clark & Rowden, 2009; Clark et al., 2015a; Clark et al., 2015b), scleractinian corals in deep, cold-water environments are predicted to be affected by global change, such as OA, much sooner than corals in surface waters of more temperate regions (Guinotte et al., 2006; Turley, Roberts & Guinotte, 2007; Sweetman et al., 2017). By 2100, under the high CO_2_ RCP8.5 scenario (IPCC, 2013), pH reductions of >0.2–0.3 pH units from current levels are expected in 23% of deep-sea canyon regions and on 8% of seamounts, the key areas where deep-water corals are typically found (Gehlen et al., 2014). While the response of deep-sea corals to OA, and resulting low carbonate saturation levels, is poorly understood, research such as that by Thresher et al. (2011), and research investigating carbonate saturation horizons in New Zealand waters by Bostock, Mikaloff Fletcher & Williams (2013), indicate that deep-sea corals will be sensitive to such environmental changes. A recent synthesis assessed the potential threat posed by OA to the diversity and productivity of New Zealand marine ecosystems, including corals, and highlighted the knowledge gaps in understanding the impacts (Law et al., 2018).

The skeletons of deep-sea scleractinian corals are most commonly composed of aragonite, the more soluble polymorph of carbonate, which makes them vulnerable to OA-induced dissolution (Anthony et al., 2008). The water’s suitability for carbonate deposition is determined by the carbonate saturation state (Ω). As Ω reduces, the formation of carbonate skeletons becomes increasingly difficult, and the increased energy requirements of calcification can ultimately threaten an organism’s survival. The depth at which seawater is saturated with aragonite is termed the aragonite saturation horizon (ASH). Below this depth, the ocean is under-saturated with respect to aragonite. From studies of distribution (e.g., see Bostock et al., 2015), it is suggested that most deep-sea coral species can probably tolerate some aragonite undersaturation (Ω_Ar_ ∼0.8–0.9). These authors suggested that scleractinian corals should be present in >1% of stations down to 1,800 m water depth, and that some species (e.g., *Solenosmilia. variabilis*) may be tolerant of Ω_Ar_ ∼0.07, but they concluded it is unclear how deep-sea corals might respond to future OA.

The rapid shoaling of the ASH over the last two decades, measured at 1–2 m yr^−1^ (Feely et al., 2012), represents a significant threat to deep-sea corals, as it is anticipated it will become challenging for these ecosystem engineers to construct and maintain their skeletons in water under-saturated with respect to aragonite (Guinotte et al., 2006; Tracey et al., 2013; Bostock et al., 2015). Globally, more than 70% of the present deep-sea coral communities will be subject to under-saturated conditions by the end of this century (Guinotte et al., 2006). However, the models used to simulate past and future changes in OA have the largest uncertainties in the Southern Ocean (e.g., Bopp et al., 2013; Orr et al., 2005). Within the New Zealand region, 95% of the habitat-forming scleractinian corals are found above the ASH (Tracey et al., 2013; Bostock et al., 2015). Recent work suggests that, during the present Anthropocene, the ASH has already shoaled by 50 to 100 m over much of the New Zealand Exclusive Economic Zone (Mikaloff-Fletcher et al., 2017). This indicates that the proportion of the region with a carbonate chemistry favourable to aragonitic calcifiers has already shrunk considerably (Mikaloff-Fletcher et al., 2017). Further, Bostock et al. (2015) noted that some scleractinian corals lie below the ASH (i.e., in a zone where conditions seem unfavourable for their growth). These authors hypothesised that previous shifts in the ASH could explain this unexpected result; corals could have established when the ASH was deeper and the waters were supersaturated with aragonite at that depth, and then adapted as the ASH shoaled (Mikaloff-Fletcher et al., 2017). Alternatively, the pattern could indicate that these corals have some capacity to withstand or acclimate to changes in ocean chemistry.

Globally, most experimental work on the effects of OA on corals has been on shallow water species and many studies note significant negative responses to OA. In meta-analyses that included studies of shallow water corals, Kroeker et al. (2010) and Kroeker et al. (2013) highlighted corals as one of the more vulnerable groups to OA. For example, calcification rates may decrease and carbonate dissolution rates may increase in shallow-water Pacific corals when pH is reduced only slightly (pH 7.85–7.95), with substantial impacts when the pH is reduced to 7.60–7.70 (Anthony et al., 2008). This pattern of decreasing calcification rates at lower carbonate concentrations is widely observed in shallow-water corals (Marubini et al., 2008; Herfort, Thake & Taubner, 2008). Cellular level effects on shallow water corals have also been observed, where the photosynthetic activity of the endosymbiont is tightly coupled with the ability of the host cell to recover from cellular acidosis after exposure to OA (Gibbin et al., 2014).

In contrast, there are fewer studies on the impacts of OA on deep-sea corals. A synthesis by Maier, Weinbauer & Gattuso (in press) reports that the response of only five deep-sea coral species (*Madrepora oculata, Lophelia pertusa, Desmophyllum dianthus, Dendrophyllia cornigera* and *Caryophyllia smithii*) to OA have been investigated. Most of the stony coral studies outside of the Mediterranean region have been confined to one species: *L. pertusa* (see Table 1 in Maier, Weinbauer & Gattuso, in press). These studies have examined effects of OA (through manipulation of pH or partial pressure of CO_2_ (pCO_2_)) after short (∼24 h) and long term (∼10–12 months) exposure, on measures such as calcification, metabolism and skeleton strength (Maier et al., 2009; Hennige et al., 2014; Movilla et al., 2014). Here we took a long-term approach (12 months) to assess the impacts of the projected end-of-century OA scenario on the physiology of an abundant, habitat-forming scleractinian coral species (*Solenosmilia variabilis*) from New Zealand and the wider southeast Pacific region (Tracey et al., 2011; Thresher et al., 2011). This species is fragile, long-lived, and late to mature (Thresher et al., 2011; Fallon, Thresher & Adkins, 2014; H Neil, DM Tracey, DM Tracey, P Marriott & MC Clark, 2010, unpublished data), and any negative impact of OA on this species could have wider ecosystem consequences.

## Methods

### Live sampling of *Solenosmilia variabilis*

Field sampling of protected corals was approved by the Department of Conservation (permit number: 35099-CAP) and coral samples were landed under the authority of the Ministry for Primary Industries (permit number: B2014/61361).

**Figure 1:**
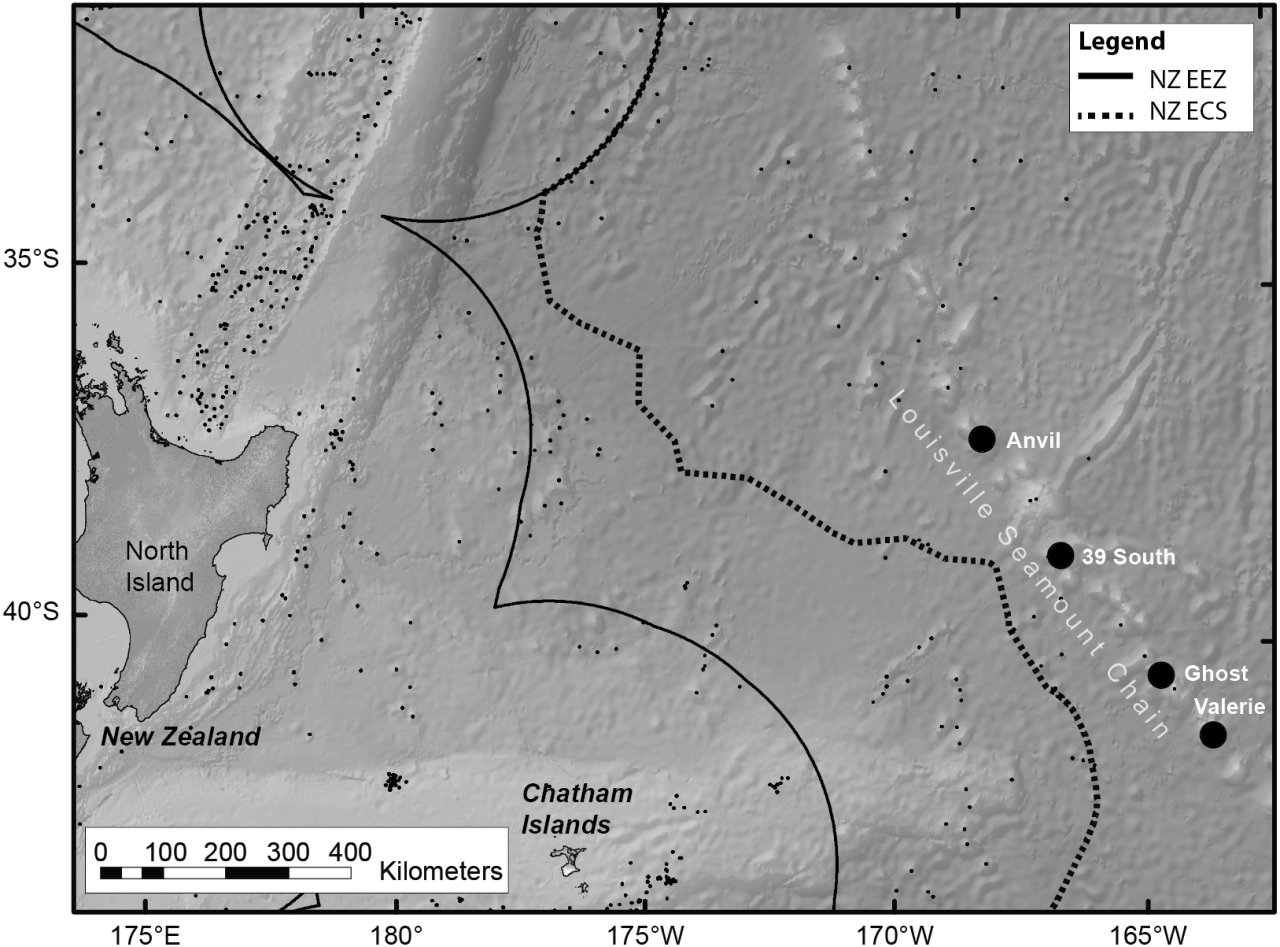
Map of the *RV Tangaroa* voyage track, within New Zealand. The map shows the Louisville Seamount Chain (named black dots), known seamount features in the region (small black dots), the Exclusive Economic Zone (EEZ) boundary and the Extended Continental Shelf (ECS) boundary. Live colonies of *Solenosmilia variabilis* were sampled using an epibenthic sled from four seamount features (Anvil, 39 South, Ghost and Valerie.). The experiment used samples from Anvil and Valerie.

Live colonies of *S. variabilis* were sampled during March 2014 from the Louisville Ridge, 700 km east of New Zealand (Fig. 1). Colonies were sampled in depths ranging from 1,220 to 1,370 m from each of two seamount-like guyot features (referred to as seamounts throughout) (Table 1) using an epibenthic sled deployed from the National Institute of Atmospheric Research (NIWA) research vessel RV *Tangaroa*.

**Table 1:** A summary of data for the Louisville Ridge sample sites, successfully sampled for live coral colonies using the epibenthic sled. The table presents the sample station depth range (m), bottom temperature (°C), and position (latitude and longitude). The pH at both sample sites was 7.88 (calculated from measured *C*_*T*_, *A*_*T*_, temperature and salinity).

Seamount	Depth range (m)	Bottom temperature (°C)	Latitude (S)	Longitude (W)	pH (calculated)
Anvil	1,244–1,370	3.42	37°42.44′	169°0.9′	7.88
Valerie	1,220–1,250	3.30	41°21.88′	164°25.14′	7.88

Upon retrieval of the sled, multiple live coral colonies were immediately placed in a chilled bin of seawater and then transferred to an on-board aquarium with a continuous flow-rate (∼50 L h^−1^) of unfiltered seawater, maintained at ∼5 °C. No feeding took place throughout the three-week voyage as it was assumed that the corals would obtain sufficient food from unfiltered surface water.

Conductivity, Temperature and Depth (CTD) casts (Seabird 911: Seattle, WA, USA) and water samples were conducted at the sampling sites in order to characterise local seawater and to inform the experimental conditions (Table 1). One CTD cast was taken per site, and the following water samples (one per site): 500 ml for total alkalinity (A_T_) and 250 ml for dissolved inorganic carbon (*C*_T_). Water samples were preserved with mercuric chloride (HgCl_2_). The *C*_T_ was determined using coulometric analysis of the CO_2_ stripped from the seawater sample after acid addition (Dickson, Sabine & Christian, 2007). The accuracy of the method is determined by analysis of Certified Reference Material (provided by Andrew Dickson from Scripps Institution of Oceanography) with every sample batch, and is estimated to be ± 1 µmol kg^−1^. A_T_ was determined using a closed cell potentiometric titration (Dickson, Sabine & Christian, 2007). The accuracy of the method is determined by analysis of Certified Reference Material (provided by Andrew Dickson from Scripps Institution of Oceanography) with every sample batch, and is estimated to be ± 2μmol kg^−1^.

In situ pH (total scale) was calculated using measured *C*_T_, A_T_, temperature and salinity, and Mehrbach equilibrium constants refit by Dickson & Millero (1987). This calculated pH was used to set the ambient pH conditions for the experiment.

### Experimental set up

Once ashore, 12 coral colonies were transferred to NIWA’s Marine Environmental Manipulation Facility (MEMF), Wellington, where they were held in flow through seawater at the temperature measured at the collection site (3.5 °C). After a stabilisation period, the large colonies were carefully broken into small portions to achieve 54 colony fragments comprising live polyps and adjacent branchlets. The number of fragments achieved from each colony ranged from two, up to six. Each colony was kept out of the water for no longer than 1 minute during this process. Each fragment (∼4–6 cm in length) was then attached in a fixed orientation to a piece of plastic mesh. Colonies were then randomly assigned to one of 18 identical tanks (4 L) with three coral fragments per tank, whilst also ensuring that fragments originating from the same colony were not included in the same tanks. Corals were maintained in darkness throughout the stablisation and experimental period.

The tanks were fed seawater via a continuous flow-through system (∼130 mL m^−1^). Seawater pH was 7.88, to mimic conditions measured at the collection sites (Table 1). The corals were fed twice weekly with a 3 mL mixture consisting of 10% commercial coral food (JBL Koralfluid, Neuhofen, Germany) and 10% commercial shellfish diet (larval shellfish diet 1800, Reed Mariculture, Campbell, CA, USA), that was diluted to the required volume with 1 µm filtered seawater (FSW). Corals were maintained in this tank set-up, with regular feeding, for three months before the experiment began, increasing the likelihood that each colony had a similar nutritional status at the beginning of the experiment.

**Table 2:** Experiment seawater conditions. pH, pCO_2_ and carbonate parameters (average ± SE) calculated from measured pH, alkalinity, temperature and salinity on two separate dates during the experiment. The pH over the entire 12 month experiment averaged 7.88 ± .00004 (control) and 7.65 ± .00007 (treatment).

Treatment (target)	pH	*A*_*T*_ (µmol kg ^−1^)	*pCO*_2_	Ω_*Ar*_	Ω_*Ca*_
pH 7.88 (control)	7.87 ± 0.0004	2,257 ± 28.71	591.9 ± 7.04	1.11 ± 0.02	1.76 ± 0.03
pH 7.65 (treatment)	7.65 ± 0.001	2,260 ± 27.51	1,017.5 ± 15.71	0.69 ± 0.01	1.09 ± 0.01

After three months, the experiment was initiated, with nine control tanks and nine treatment tanks established. Corals in the control group were exposed to ambient pH 7.88 (pCO_2_519 ppm). In comparison, treatment corals were exposed to low pH of 7.65 (pCO_2_920 ppm) (Table 2). The reduced pH level was based on projected changes to seawater pH through to the year 2100 (Bopp et al., 2013; IPCC, 2013; Orr et al., 2005). The pH in the treatment tanks was reduced gradually over three days until it reached the treatment value. Temperature was held at 3.5 °C in all tanks.

### Seawater manipulation and measurement

FSW from Wellington Harbour, adjacent to the facility, was chilled to 3.5 °C and fed to separate header tanks before being delivered to the experimental tanks at 130 mL min^−1^ in a flow-through system. The pH was adjusted through the diffusion of food grade CO_2_, which was controlled using Sensorex S150C pH probes (Garden Grove, CA, USA). The pH probes in each header tank were calibrated regularly with TRIS and AMP buffers. Water samples were taken from each header tank on two occasions during the 12-month experiment, preserved with HgCl_2_, and analysed for determination of *A*_*T*_ as described above. These measurements of pH (on each day the water samples were taken) and *A*_*T*_, along with temperature and salinity, were used to calculate pCO_2_ and Ω_Ar_ of each experimental treatment using the refitted (Mehrbach et al., 1973) equilibrium constants (Dickson & Millero, 1987).

### Evaluating *Solenosmilia variabilis* responses

Responses were assessed using a variety of measures at regular intervals over the 12-month experiment. At the beginning of the experiment, all coral fragments were photographed and buoyant weighed. Subsequently, at bimonthly intervals over a 10-month period, measurements of polyp mortality (via live polyp counts), linear skeletal extension and / or three-dimensional step-wise growth, (referred to as linear growth throughout), and loss of coenenchyme tissue were made. The coenenchyme is the outer tissue covering the coral skeleton, that links the coral polyps and provides protection for the developing exoskeleton; loss of this tissue was evaluated via changes in colour saturation (detailed below). Respiration rate (O_2_ consumption) was measured on two occasions, at six and 12 months.

### Polyp mortality

Polyp mortality was measured every two months by making a visual count of the number of live polyps on each fragment. Each tank had three fragments and polyp mortality was averaged for each tank to get a single average per tank (*n* = 9). The total percentage remaining of the initial polyp count at each time point was then calculated using the following equation:

\begin{eqnarray*}100- \left[ \left( \frac{ \left( {P}_{1}-{P}_{J} \right) }{ \left( {P}_{1} \right) } \right) \right] \times 100 \end{eqnarray*}

Where *P*_1_ is the polyp count taken at the first time point and *P*_*J*_ is the polyp count at each of the subsequent *J*th time points.

### Linear growth

Each coral fragment was photographed at bimonthly intervals to obtain a measure of linear growth. Because fragments were cable-tied in a fixed position, they remained in the same orientation throughout the experiment, and it was possible to locate and measure the same branch through time. From the digital images, linear growth was determined by selecting an easily identifiable feature on the colony fragment, such as a branching point or a specific linear growth feature. Measurements were then taken from this distinctive point, along the axis of linear growth, to the area just below a live polyp where the calcification process occurs. Measurements were made using the software *ImageJ* © (Schneider, Rasband & Eliceiri, 2012). Where possible a maximum of four such measurements were taken for each fragment. Where multiple measurements were taken, these were then averaged to achieve a single linear growth rate for each fragment. Each tank had three fragments and the single fragment linear growth rates were averaged for each tank to get a single average per tank (*n* = 9). Only branchlets that were ∼2–5 cm long at the beginning of the experiment were selected for measurement.

### Tissue loss

Images taken to measure linear growth rates were also analysed to determine colour saturation, which was used as a proxy for the coenenchyme covering the branch and polyp areas of the coral skeleton. Our method used to assess colour change was based on that of Winters et al. (2009). Images taken during the experiment were cropped to remove the background and then colour-profiled using the *colour histogram* plugin on *ImageJ*. The entire 2D image of each coral fragment was profiled at each time point. This profile provides a mean value of intensity for each of the red, green and blue colour channels. A pilot study was used to confirm that a loss in intensity of the red colour channel corresponded to a loss of coenenchyme (see Supplementary Information).

The relative intensity for the red colour channel was calculated using the following equations:

\begin{eqnarray*}& & T=R+G+B \end{eqnarray*}

\begin{eqnarray*}& & {R}_{r}= \frac{R}{T} \end{eqnarray*}

\begin{eqnarray*}& & {G}_{r}= \frac{G}{T} \end{eqnarray*}

\begin{eqnarray*}& & {B}_{r}= \frac{B}{T} \end{eqnarray*}

where T, the total intensity of an image; R, mean intensity of the red channel; G, mean intensity of the green channel; B, mean intensity of the blue channel; and *R*_*r*_, *G*_*r*_ and *B*_*r*_, relative intensity of the red, green and blue channels, respectively (Winters et al., 2009). Calculating the percentage of relative brightness for the red colour channel, rather than using the mean brightness, suppresses the influence that any changes in illumination, exposure or internal camera processing, may have on the brightness of each channel (Richardson et al., 2009).

The percentage change in relative intensity of the red colour channel was then calculated using the following equation:

\begin{eqnarray*}{R}_{R}=100\times \frac{ \left( {S}_{R1}-{S}_{R2} \right) }{ \left( {S}_{J1}\mathrm{x} \frac{{T}_{1}}{{T}_{2}} \right) } \end{eqnarray*}

where *R*_*R*_, the relative intensity of the red colour channel; *S*_*R*1_, the mean intensity of the red colour channel at time point one; *S*_*R*2_, the mean intensity of the red colour intensity at time point two; *T*_1_, time point one and *T*_2_ time point two.

### Respiration rate

**Figure 2:**
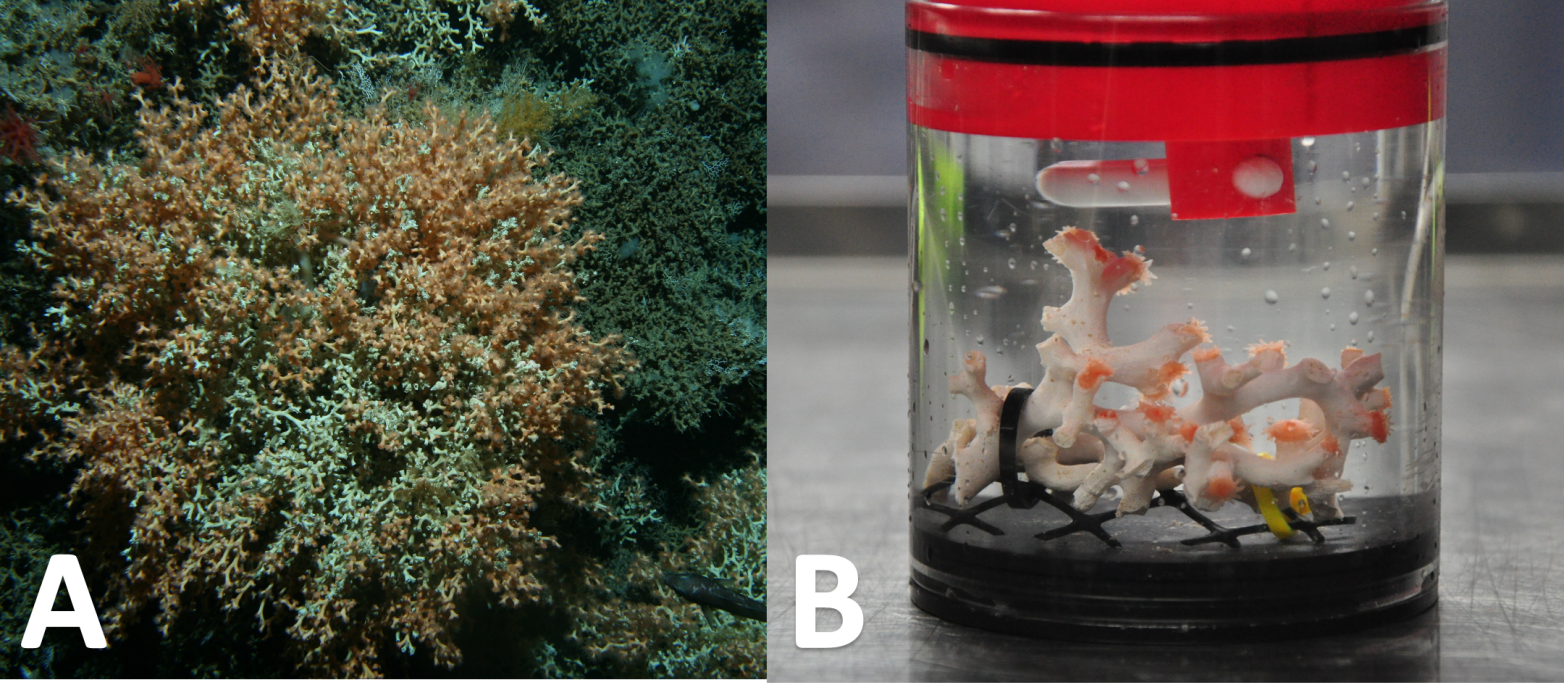
*Solenosmilia variabilis* colony in situ and *S. variabilis* fragment in a respiration chamber. The images show a large colony of deep-sea coral *Solenosmilia variabilis* on a seamount flank in the New Zealand region (A) (NIWA Deep Towed Imaging System); and a fragment of *S. variabilis* in the experimental respiration chamber (B), a stirrer is positioned at the top of the chamber. (This figure is derived in part from an article published in *the New Zealand Journal of Marine and Freshwater Research* published on 25 September 2017, available online: https://doi.org/10.1080/00288330.2017.1374983).

At six and 12 months, one fragment per tank was randomly selected (*n* = 9 for each treatment and time point), and respiration rates measured. Respiratory oxygen consumption was measured in a 500 mL chamber, sealed by an o-ring (Fig. 2).

Each chamber was equipped with a magnetic stirrer to ensure homogeneity of oxygen (O_2_) around the coral fragments. A glass vial with a Presens Pst 3 O_2_ sensor (Regensburg, Germany) glued to its end was inserted through a hole in the chamber lid, so that it made contact with seawater in the chamber. The O_2_ sensor was two-point calibrated before each run, using 0% and 100% saturated seawater; 0% saturated seawater was obtained by dissolving 1 g of sodium sulphite (Sigma-Aldrich, St. Louis, MO, USA) in seawater, and 100% saturation was achieved by bubbling air through seawater for 30 min. The chambers were placed in a 3.5°C water bath and kept in darkness. Each coral fragment was left to settle in its chamber for a minimum of 20 min before the chamber was sealed. The chambers remained in the water bath for the duration of the measurement and were kept in darkness to prevent any photosynthetic activity in the seawater. Total O_2_ used by each coral fragment was calculated as the difference between the initial and final oxygen concentrations measured within each chamber.

Each run consisted of five incubation chambers, each housing a different coral fragment. The duration that each fragment was kept in a chamber varied depending on the coral’s respiration rate, a period ranging from 5 to 7 h. Measurements in the chambers were terminated if the O_2_ saturation dropped below a pre-determined 90%. The water volume within each chamber was measured at the end of each experiment.

The O_2_ concentration in each chamber at the start and end of the experiment was standardised to µg L^−1^ and an hourly rate of O_2_ consumption calculated for each individual. The protein content *per* individual was used to normalise the respiration rate (µg O_2_ mg protein^−1^ h^−1^). Samples were initially frozen and the frozen tissue removed from the skeleton matrix with an airbrush and transferred to a snap-lock bag containing 5 mL of distilled water. The protein slurry produced was then poured into a 250 mL beaker. The snap-lock bag was rinsed into the beaker twice with 5 mL of distilled water to remove any residual protein. The protein slurry was homogenized further using an electric homogenizer (Proxxon micropower driver, Föhren, Germany), and the total quantity of homogenized material noted. A 5 µL subsample of homogenized protein slurry was transferred to a 96-well plate and analysed with the Coomassie Brilliant Blue protein assay (Bradford, 1976), and a spectrophotometer (EnSpire 2300 Multilabel Plate Reader: PerkinElmer, Waltham, MA, USA). The protein concentration of each 5 µL sub-sample was then adjusted for the total volume of each sample and the total protein content of each individual coral fragment calculated.

### Statistical analyses

Statistical analyses were carried out using the software package SPSS (Coakes & Steed, 2009). Data were initially tested for normality, and transformed if they did not meet assumptions. A Friedman test was used to analyse data for both polyp mortality and the loss of coenenchyme (data were not normally distributed, and the assumption of normality could not be met using log transformations). Data were categorized into 12 groups which represented each of the monthly time points (zero, two, four, six, eight and 10 months of exposure) for the treatment and control pH samples.

A rm-ANCOVA was used to compare the average linear growth rate of individuals between the control and treatment groups. The difference between the linear growth lengths of each branchlet, for each sample, was compared between each time point and linear growth presented as mm linear extension *per* year. Seamount and colony of origin were included as covariates to ensure that they had no confounding effects on the response variable.

Respiration data were log transformed to meet the assumption of normality. The significant effect of treatment, and interactive effect of time since exposure with treatment, were tested using a two-way ANCOVA. Respiration chamber, seamount of origin and colony of origin were included as covariates to ensure that they had no confounding effects on the response variable.

## Results

At the end of the 12-month experiment, all corals, in both the treatment and control groups, had live polyps, indicating that the experimental conditions were appropriate to maintain viable corals.

pH was maintained at target concentrations for the duration of the experiment. The average pH for the treatment group was 7.650 ± .00007 (range: 7.604–7.699), and the average pH for the control group was 7.876 ± .00004 (range: 7.823–7.920). These averages are calculated from >2,800 pH probe measurements, taken throughout the 12-month experiment.

### Polyp mortality

A visible increase in polyp mortality was noted throughout the experiment. No polyp mortality occurred in the first two months in either the control or treatment groups, although it subsequently increased over time. While there was a treatment effect (Friedman test, *χ*2(11) = 107.769, *p* = 0.001), post hoc tests (Wilcoxon-signed rank test) showed that these differences were between different time points of the same treatment and that there was no change in polyp mortality within the treatment group, relative to the control. However, from six months onward, polyp mortality was consistently higher in the low pH group. The greatest loss in polyp mortality occurred in the low pH group from four (where colonies still had 92.04% ± 7.45 of their polyps remaining) to six months of exposure (where colonies only had 61.58% ± 7.19 of their polyps remaining). The difference between these two time points represents a loss of 30.46% of initial polyp counts over just a four-month period.

### Linear growth

The average linear extension rate at the control pH was 1.558 ± 0.226 mm year^−1^ and at the reduced pH was 1.603 ± 0.260 mm year^−1^. Linear growth rates were highly variable between individual coral fragments, ranging from 0.583 to 3.068 mm year ^−1^.

Linear growth rate was also independent of time of exposure for both the reduced pH and control groups (rm-ANCOVA, *F*_4,40_ = 0.481, *p* = 0.749 and *F*_4,52_ = 0.274, *p* = 0.893, respectively). The seamount of origin also had no effect on the linear extension rate of the treatment colonies (rm-ANCOVA, *F*_4,40_ = 0.769, *p* = 0.552) or the control colonies (*F*_4,52_ = 0.577, *p* = 0.681). For these reasons, both time of exposure and seamount were excluded from the final analyses, which then found no effect of reduced pH on the linear extension rate of *S. variabilis* (rm-ANCOVA, *F*_1,25_ = 0.017, *p* = 0.899).

### Coenenchyme loss

**Figure 3:**
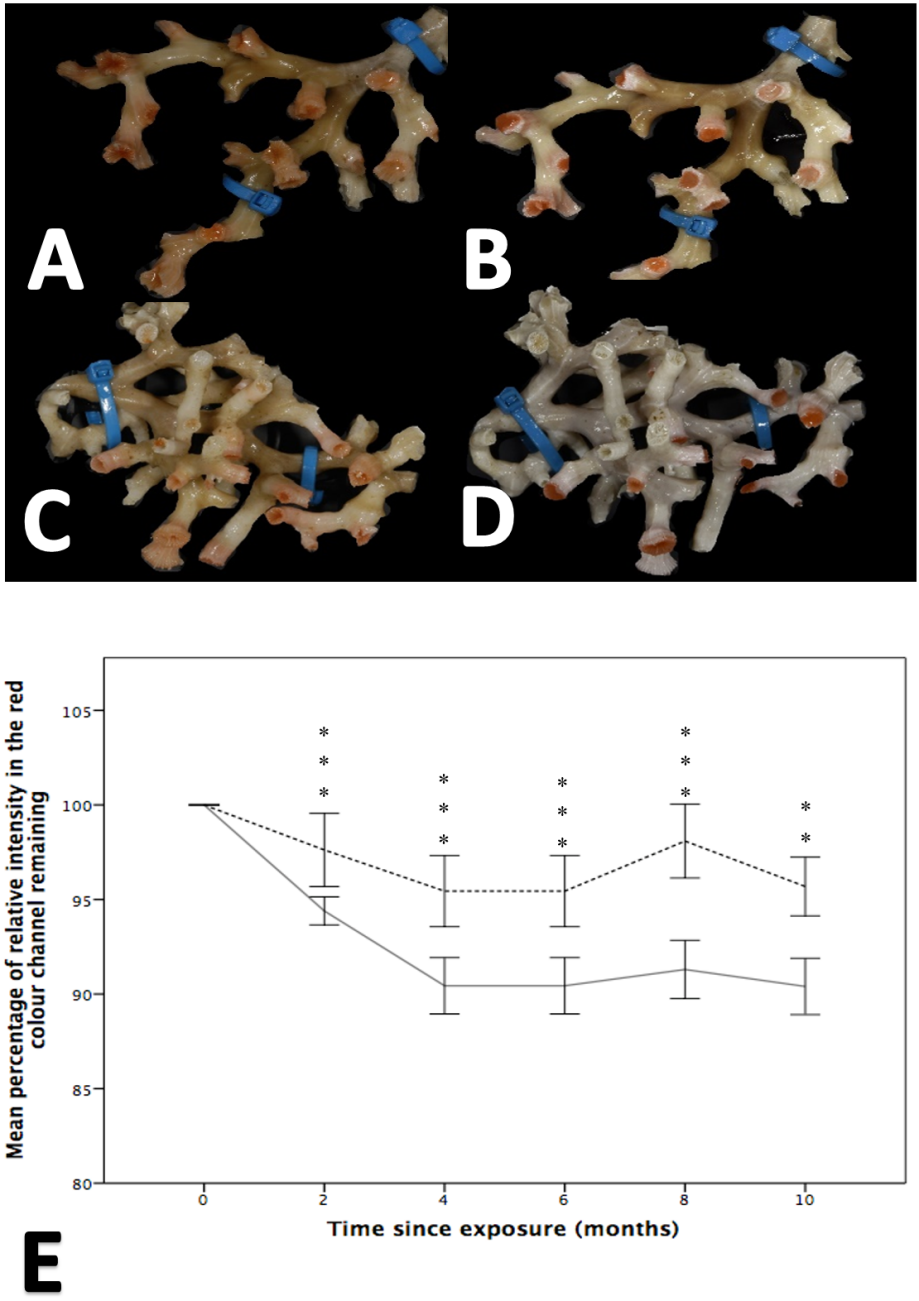
The effect of seawater pH on the loss of coenenchyme tissue of the coral *Solenosmilia variabilis*. Images A–B are of the same colony at control pH (pH 7.88). Images C–D are of the same colony at the treatment pH (pH 7.65). Images A and C were taken prior to the start of the experiment, and images B and D after three months into the experiment. Note the marked reduction in the intensity of the red/pink colouration to a pale colour once the coral had been exposed to low pH for several months (D). Photos of *S. variabilis* were analysed for the relative percentage of intensity in the red colour channel (*n* = 17 per time-point per treatment). The mean percentage remaining (± 1 SE) of the initial relative intensity is presented (E). The solid line represents the treatment group and the broken line represents the control group. Significant differences from the control are shown by **p < 0.01 and ***p < 0.001 (Wilcoxon-signed rank analysis).

While both the control and treatment groups lost colour throughout the experiment, colour loss was significantly greater in fragments held at reduced pH, a finding that was apparent at all time points (i.e., 2, 4, 6, 8 and 10 months; Wilcoxon-signed rank analysis post hoc analysis, Friedman test, *χ*2(11) = 130.617, *p* = 0.001; Fig. 3). After two months, the colour intensity of the control group was 97.61% ± 1.933 of that measured at the start of the experiment, while the low pH group retained 94.396% ± 0.738 of its colour intensity. By comparison, at 10 months there was, on average, a difference of 5.28% between the percentage of initial colour remaining between the treatment and control groups.

### Respiration rate

Coral respiration rates were higher in the control pH than in the low pH at both the six and 12-month time points (Fig. 1) and, for all fragments, were higher at the 12-month time point. For fragments in the control group (pH 7.88), respiration was 179% and 31% higher than for coral colonies exposed to low pH (pH 7.65) after six and 12 months, respectively (Fig. 1). Also of note is that the respiration rates at reduced pH increased by 225% between the six and 12-month time points.

**Figure 4:**
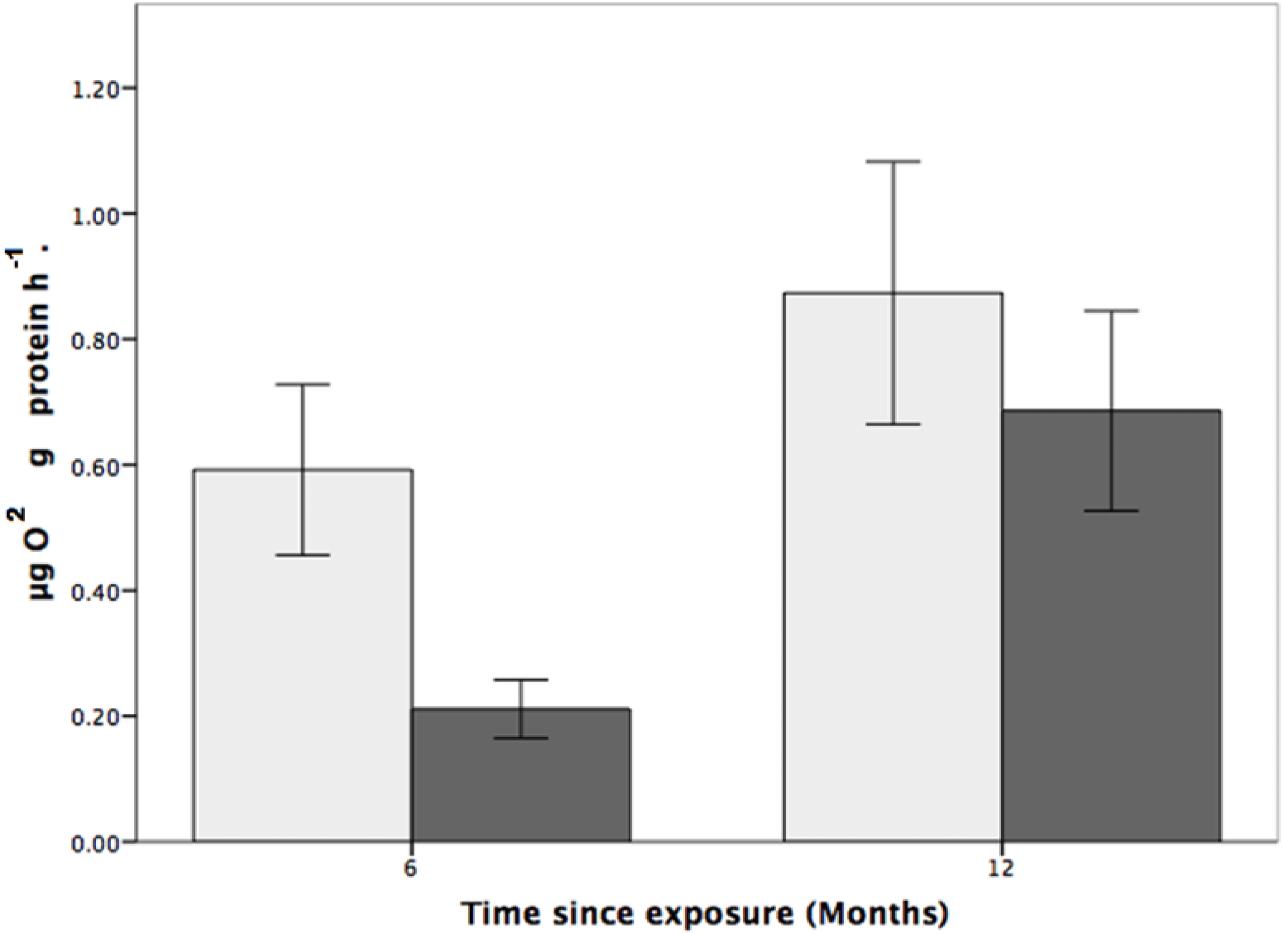
The effects of seawater pH on the respiration rate of *Solenosmilia variabilis*. Respiration rate (µmol O_2_ mg protein^−1^ h^−1^) of colonies after exposure to reduced pH (pH 7.65; dark grey) or control pH (pH 7.88; light grey) for six- and 12-month exposure. (*n* = 9 for each treatment and time-point; values are means ± standard error (SE)).

While the statistical analyses indicated that respiration rate was not influenced by pH (two-way ANOVA, *F*_1,24_ = 3.200, *p* = 0.086; Fig. 4), there was a significant effect of time, where respiration rates were higher, for both the control and treatment groups, at the 12-month time point (two-way ANOVA, *F*_1,24_ = 0.977, *p* = 0.007). There was, however, no interactive effect between pH treatment and time (two-way ANOVA: *F*_1,24_ = 0.101, *p* = 0.350).

## Discussion

This study investigated physiological responses to reduced pH in *S. variabilis*, a habitat-forming scleractinian coral species common around New Zealand and the wider southeast Pacific region.

*S. variabilis* colonies were maintained for 12 months under reduced pH conditions (pH 7.65, Ω_Ar_ = 0.69 ± 0.01), and various aspects of their physiological response were investigated over that time. While the colonies were generally robust to OA conditions (there was no mortality), there was significant loss of coenenchyme tissue cover at low pH (Fig. 3), and indications of effects on respiration rates (Fig. 4). Respiration rate was relatively low in the reduced pH treatment, particularly at the six-month time point when it was 179% higher in control conditions (Fig. 4), although this effect was not statistically significant. There was no treatment effect on mortality of polyps or linear growth rates.

To date, published studies on how OA might influence deep-sea corals have varied results, even within different populations of the same species. This is demonstrated by Georgian et al. (2016) who tested the physiological response to OA of *L. pertusa* colonies from two geographically different populations (Gulf of Mexico, USA and Tisler Reef, Norway). The Gulf of Mexico corals exhibited reductions in net calcification and respiration, while Tisler Reef corals showed only slight reductions in net calcification and elevated respiration. The authors concluded that these differences were likely the result of environmental differences (e.g., depth, pH, food supply) between the two regions. In another experiment on *L. pertusa,* Maier et al. (2009) found that incubating *L. pertusa* for 24 h in seawater with pH lowered by 0.15 and 0.3 units, relative to the ambient level, resulted in calcification being reduced by 30 and 56%, respectively. In another short term study, Hennige et al. (2014) investigated the response of *L. pertusa* to increased CO_2_ conditions (750 ppm) over 21 days. *L. pertusa* corals exposed to increased CO_2_ had significantly lower respiration rates than corals in control conditions, but found no corresponding change in calcification rates. In a longer-term study, Movilla et al. (2014) found a decline in the calcification of *D. dianthus* after 314 days of exposure to elevated pCO2 (800 µatm). Interestingly, in another long-term experiment, over 12 months, Hennige et al. (2015) observed a decrease in the structural integrity of dead exposed *L. pertusa* skeleton, when exposed to increased CO_2_ conditions. Such studies provided a platform for our current study.

The Ω_Ar_ was <1 in both the control and treatment waters in our experiment (Table 2). From a broad survey of New Zealand coral species and carbonate saturation, Tracey et al. (2013) and Bostock et al. (2015) identified a strong dependency of coral distribution on Ω_Ar_ and Ω_Ca_. However, many deep-sea stony corals can cope with some degree of aragonite undersaturation (Ω_Ar_ ∼0.8–0.9) with some species tolerant of Ω_Ar_ ∼0.7 (Bostock et al., 2015), a value lower than the Ω_Ar_ of 0.69 ± 0.01 in our low pH treatment. While it has been noted that some stony corals lie below the ASH (e.g., Bostock et al., 2015; Baco et al., 2017), including in the New Zealand region, such as those found along the Louisville Seamount Chain (Bostock et al., 2015), we did note reduced coenenchyme tissue cover, and indications of elevated respiration rates at these levels.

### Linear growth rates

This study found no treatment effect on the linear growth rates of corals which were seen to be highly variable, ranging from 0.583 to 3.068 mm y^−1^. The measured linear growth rate is comparable to results from radiocarbon dating studies of *S. variabilis* by Fallon, Thresher & Adkins (2014), who reported linear growth-rates ranging between 0.84–1.25 mm y^−1^, and by H Neil, DM Tracey, DM Tracey, P Marriott & MC Clark (2010, unpublished data), who again showed similar linear growth of 0.25–1.3 mm y^−1^. Linear growth rates that are independent of pH have been found in other deep-sea corals from various *in aquaria* studies, including *M. oculata* (Maier et al., 2013b) and *L. pertusa* (Form & Riebesell, 2012; Maier et al., 2013b). While it was found that *M. oculata* was not affected when pH was manipulated to end-of-century projections, when the partial pressure of CO_2_ was reduced to pre-industrial levels, calcification rates in this species increased (Maier et al., 2012). This provides important information about the historical effect of OA on the calcification of deep-sea corals and indicates that the present-day calcification rates may have already declined due to an anthropogenic increase in the concentration of atmospheric CO_2_. Although no net effect of OA on linear extension was observed in this study, it is important to note that measurements were only taken after several months and undetected shorter-term changes may have occurred.

### Respiration rate

Respiration rate was highly variable, ranging from 0.065 µg O_2_ g protein^−1^ h^−1^ to 1.178 µg O_2_ g^−1^ protein^−1^ h^−1^. These results are low compared to respiration rates found by Dodds et al. (2007) for the branching scleractinian *L. pertusa*, who found a respiration rate of about 0.5 µmol g^−1^ h^−1^. Here, the respiration rate of *S. variabilis* was relatively low in the reduced pH treatment, particularly at the six-month time point. Interestingly, this difference decreased at the 12-month time point, and this could be indicative of acclimation. A similar response was found by Maier et al. (2013a) in *M. oculata* and *L. pertusa*. The authors attributed the observed increase in respiration rate to an increase in energy supply as a result of regular feeding, thus sustaining an elevated level of coral metabolism. Regular feeding, and its impact on coral metabolism, can mask the effects of OA in experimental work. This was found by Büscher, Form & Riebesell (2017) who concluded that, while the deep-sea coral, *L. pertusa,* is capable of calcifying under elevated CO_2_ and temperature, its condition (fitness) is more strongly influenced by food availability rather than changes in seawater chemistry. With the natural habitat of *S. variabilis* being so inaccessible, it is difficult to predict the amount of food, including particulate organic matter and sources of plankton, reaching colonies in situ.

### Tissue loss

A visible loss of coenenchyme was noted from both the control and treatment colonies in the first two months of the experiment, although this loss was significantly greater from the treatment colonies. Tissue loss in the control group is consistent with stress and colony deterioration, which is not surprising given that deep-sea corals are difficult to maintain in a healthy state *in aquaria*; indeed, to our knowledge, *S. variabilis* has never previously been maintained for more than a few weeks in this state. The significantly greater effect of reduced pH on the rate of tissue loss highlights that other physiological mechanisms were also playing a part. A loss of tissue when exposed to OA conditions has also been observed in tropical corals (e.g., *Pocillopora damicornis* and* Oculina patagonica*), but the tissues of these two species regenerated when the corals were returned to ambient pH (Kvitt et al., 2015). For these shallow, warm-water corals, reduced pH induced tissue-specific apoptosis, a breakdown of coenenchyme and a subsequent loss of the colonial form. The tissue loss of *S. variabilis* seen here could represent the early stages of a similar response and warrants a longer-term study. Interestingly, the same rate of polyp mortality over time was observed in both the treatment and control colonies, while coenenchyme loss was greater in the treatment group. The coenenchyme has a function in connecting each neighbouring polyp and protecting the growing skeleton. A loss of the coenenchyme could mean a shift away from the coral’s ability to produce a colonial three-dimensional matrix (Hennige et al., 2015). Reverting to solitary and non-calcifying polyps has been proposed as an evolutionary mechanism which has allowed corals to survive through geological periods of unfavourable calcification conditions (Kvitt et al., 2015), and could explain several “reef gaps” in the geological records (Wood, 1999).

Alternatively, the observed loss of coenenchyme could represent a reallocation of energy. That is, corals in the treatment group may have been diverting energy away from the maintenance of tissues, allowing them to maintain other metabolic requirements (e.g., linear growth, respiration and reproduction). For this reason, tissue loss in corals is considered a better indicator of physiological stress than skeletal linear growth (Anthony, Connolly & Willis, 2002). Maier et al. (2016) show that the energy required for calcification in *M. oculata* is a small fraction (∼1-3%) of overall metabolic requirements. Assuming that the energy requirements for calcification in *S. variabilis* are similar, this substantiates our comment that tissue loss may be a better indicator of physiological stress than linear growth and partly explains why this study found no treatment effect on the linear growth rates of corals.

## Conclusion

Deep-sea corals are typically difficult to study due to their poor survival rate in laboratory conditions. For this reason physiological studies of their responses to environmental change have been limited to date. The data presented here for *S. variabilis* represent an important first-step towards understanding the biology of this ecologically important species, and to our understanding of the sensitivity of deep-sea corals to OA. In New Zealand specifically, the lack of knowledge of organism responses is well recognised: the potential threat posed by OA to the diversity and productivity of marine ecosystems (including to corals) is classed as medium for vulnerability, low to medium for knowledge of established response, and low for understanding mechanistic response, ecosystem interaction, and interaction of other stressors (Law et al., 2018). This study found that *S. variabilis* lost tissue in response to OA and we hypothesize that this could represent a reallocation of energy, with corals diverting energy away from the maintenance of non-essential tissue. It is assumed however, that an organism would not continue to break down tissues to help support skeletal three-dimensional linear and/or step-wise growth, as a threshold will ultimately be reached where the animal becomes seriously compromised. If this were to happen, then there would be major changes to the structure and function of this species as an important ecosystem engineer in the deep-sea.

This study has signposted the need to better understand the long-term implications and mechanisms of OA on colony tissue loss, the most notable effect of decreased pH observed. To our knowledge, this study is the first to apply a technique of measuring tissue loss to a deep sea coral in an experiment designed to measure the corals’ response to OA. Studies, such as this, which find a limited response in those physiological variables which are typically measured (e.g., respiration and linear growth), should consider what the potential cost of maintaining those parameters may be. Here we demonstrate an additional measure, of tissue loss, which could be routinely included in future studies to gain a more holistic understanding of the organisms’ response. We also recommend that future studies assess the impact of OA on skeletal morphology and density, which were not assessed here. Such impacts have the potential to change colony integrity and survival. Combined with ongoing and more refined modelling work to inform future projections of the ASH and CSH in the South Pacific, this study nevertheless improves our knowledge on the impacts of OA on this important and ecologically vulnerable coral group in the New Zealand region.

##  Supplemental Information

10.7717/peerj.5236/supp-1 Data S1 Combined data set Growth rates (mm, bi-monthly)
Polyp mortality (bi-monthly)
Coenenchyme loss (% remaining in the red colour channel)
Respiration (µmol O_2_ mg protein ^−1^ h^−1^).

10.7717/peerj.5236/supp-2 Supplemental Information 1 Supplementary information Pilot study and method development to measure the loss of coenenchyme.

## Additional Information and Declarations

### Field Study Permissions

The following information was supplied relating to field study approvals (i.e., approving body and any reference numbers): Field sampling of protected corals was approved by the Department of Conservation (permit number: 35099-CAP) and coral samples were landed under the authority of the Ministry for Primary Industries (permit number: B2014/61361).

### Data Availability

The following information was supplied regarding data availability: The raw data are provided in a Supplemental File.

## References

[ref-1] Anthony K, Connolly SR, Willis BL (2002). Comparative analysis of energy allocation to tissue and skeletal growth in corals. Limnology and Oceanography.

[ref-2] Anthony KR, Kline DI, Diaz-Pulido G, Dove S, Hoegh-Guldberg O (2008). Ocean acidification causes bleaching and productivity loss in coral reef builders. Proceedings of the National Academy of Sciences of the United States of America.

[ref-3] Baco AR, Morgan N, Roark EB, Silva M, Shamberger KE, Miller K (2017). Defying dissolution: discovery of deep-sea scleractinian coral reefs in the North Pacific. Scientific Reports.

[ref-4] Biber MF, Duineveld GC, Lavaleye MS, Davies AJ, Bergman MJ, Van den Beld IM (2013). Investigating the association of fish abundance and biomass with cold-water corals in the deep Northeast Atlantic Ocean using a generalised linear modelling approach. Deep Sea Research Part II: Topical Studies in Oceanography.

[ref-5] Bongiorni L, Mea M, Gambi C, Pusceddu A, Taviani M, Danovaro R (2010). Deep-water scleractinian corals promote higher biodiversity in deep-sea meiofaunal assemblages along continental margins. Biological Conservation.

[ref-6] Bopp L, Resplandy L, Orr JC, Doney SC, Dunne JP, Gehlen M, Halloran P, Heinze C, Ilyina T, Séférian R, Tjiputra J, Vichi M (2013). Multiple stressors of ocean ecosystems in the 21st century: projections with CMIP5 models. Biogeosciences.

[ref-7] Bostock H, Mikaloff Fletcher SE, Williams MJ (2013). Estimating carbonate parameters from hydrographic data for the intermediate and deep waters of the Southern Hemisphere Oceans. Biogeosciences.

[ref-8] Bostock HC, Tracey DM, Currie KI, Dunbar GB, Handler MR, Mikaloff Fletcher SE, Smith AM, Williams MJM (2015). The carbonate mineralogy and distribution of habitat-forming deep-sea corals in the Southwest Pacific region. Deep-sea research Part I: Oceanographic Research Papers.

[ref-9] Bradford MM (1976). A rapid and sensitive method for the quantitation of microgram quantities of protein utilizing the principle of protein-dye binding. Analytical Biochemistry.

[ref-10] Buhl-Mortensen L, Mortensen (2005). Distribution and diversity of species associated with deep-sea gorgonian corals off Atlantic, Canada. Cold-water Corals and Ecosystems.

[ref-11] Büscher JV, Form AU, Riebesell U (2017). Interactive effects of ocean acidification and warming on growth, fitness and survival of the cold-water coral *Lophelia pertusa* under different food availabilities. Frontiers in Marine Science.

[ref-12] Cairns SD (1979). The deep-sea Scleractinian of the Caribbean Sea and adjacent waters. Studies on the Fauna of Curacao and other Caribbean Islands.

[ref-13] Clark MR, Althaus F, Schlacher TA, Williams A, Bowden DA, Rowden AA (2015a). The impacts of deep-sea fisheries on benthic communities: a review. ICES Journal of Marine Science.

[ref-14] Clark MR, Anderson O, Bowden D, Chin C, George S, Glasgow D, Guinotte J, Hererra S, Osterhage D, Pallentin A, Parker S, Rowden AA, Rowley S, Stewart R, Tracey D, Wood S, Zeng C (2015b). Vulnerable marine ecosystems of the Louisville Seamount chain: voyage report of a survey to evaluate the efficacy of preliminary habitat suitability models. New Zealand aquatic environment and biodiversity Report No. 149.

[ref-15] Clark MR, Rowden AA (2009). Effect of deepwater trawling on the macro-invertebrate assemblages of seamounts on the Chatham Rise, New Zealand. Deep Sea Research Part I: Oceanographic Research Papers.

[ref-16] Coakes SJ, Steed L (2009). SPSS: analysis without anguish using SPSS version 14.0 for Windows.

[ref-17] Dickson AG, Millero FJ (1987). A comparison of the equilibrium constants for the dissociation of carbonic acid in seawater media. Deep Sea Research Part A. Oceanographic Research Papers.

[ref-18] Dickson AG, Sabine CL, Christian JR (2007). Guide to best practices for ocean CO2 measurements.

[ref-19] Dodds LA, Roberts JM, Taylor AC, Marubini F (2007). Metabolic tolerance of the cold-water coralLophelia pertusa(Scleractinia) to temperature and dissolved oxygen change. Journal of Experimental Marine Biology and Ecology.

[ref-20] Fallon S, Thresher R, Adkins J (2014). Age and growth of the cold-water scleractinian Solenosmilia variabilis and its reef on SW Pacific seamounts. Coral Reefs.

[ref-21] Feely RA, Sabine CL, Byrne RH, Millero FJ, Dickson AG, Wanninkhof R, Murata A, Miller LA, Greeley D (2012). Decadal changes in the aragonite and calcite saturation state of the Pacific Ocean. Global Biogeochemical Cycles.

[ref-22] Form AU, Riebesell U (2012). Acclimation to ocean acidification during long-term CO2 exposure in the cold-water coral Lophelia pertusa. Global Change Biology.

[ref-23] Fosså JH, Kutti T, Helle K, Bergstad OA (2012). Associations and functional links between tusk and cold water coral and sponge habitats examined by experimental long-line fishing.

[ref-24] Freiwald A, Fossa J, Grehan A, Koslow T, Roberts J (2004). Cold-water coral reefs: out of sight no longer out of mind.

[ref-25] Friedrich T, Timmermann A, Abe-Ouchi N, Bates M, Chikamoto M, Church J, Dore D, Gledhill M, Gonzalez-Davila M, Heinemann T, Ilyina J, Jungclaus E, McLeod A, Santana-Casiano JM (2012). Detecting regional anthropogenic trends in ocean acidification against natural Variability. Natural Climate Change.

[ref-26] Gehlen M, Séférian R, Jones DO, Roy T, Roth R, Barry J, Joos F (2014). Projected pH reductions by 2100 might put deep North Atlantic biodiversity at risk. Biogeosciences.

[ref-27] Georgian SE, Dupont S, Kurman M, Butler A, Strömberg SM, Larsson AI, Cordes EE (2016). Biogeographic variability in the physiological response of the cold-water coral Lophelia pertusa to ocean acidification. Marine Ecology.

[ref-28] Gibbin EM, Putnam HM, Davy SK, Gates RD (2014). Intracellular pH and its response to CO2-driven seawater acidification in symbiotic versus non-symbiotic coral cells. The Journal of Experimental Biology.

[ref-29] Guinotte J, Orr J, Cairns S, Freiwald A, Morgan L, George R (2006). Will human-induced changes in seawater chemistry alter the distribution of deep-sea scleractinian corals?. Frontiers in Ecology and the Environment.

[ref-30] Hennige SJ, Wicks LC, Kamenos NA, Bakker DCE, Findlay HS, Dumousseaud C, Roberts JM (2014). Short-term metabolic and growth responses of the cold-water coral Lophelia pertusa to ocean acidification. Deep Sea Research Part II: Topical Studies in Oceanography.

[ref-31] Hennige SJ, Wicks LC, Kamenos NA, Perna G, Findlay HS, Roberts JM (2015). Hidden impacts of ocean acidification to live and dead coral framework. Proceedings of the Royal Society B: Biological Sciences.

[ref-32] Henry LA, Davies AJ, Roberts JM (2010). Beta diversity of cold-water coral reef communities off western Scotland. Coral Reefs.

[ref-33] Henry LA, Navas JM, Hennige SJ, Wicks LC, Vad J, Roberts JM (2013). Cold-water coral reef habitats benefit recreationally valuable sharks. Biological Conservation.

[ref-34] Herfort L, Thake B, Taubner I (2008). Bicarbonate stimulation of calcification and photosynthesis in two hermatypic corals. Journal of Phycology.

[ref-35] Hoegh-Guldberg O, Mumby PJ, Hooten AJ, Steneck RS, Greenfield P, Gomez E, Hatziolos ME (2007). Coral reefs under rapid climate change and ocean acidification. Science.

[ref-36] Stocker TF, Qin D, Plattner GK, Tignor M, Allen SK, Boschung J, Midgley BM, IPCC (2013). Climate change 2013: the physical science basis. Contribution of working group I to the fifth assessment report of the intergovernmental panel on climate change.

[ref-37] Kroeker KJ, Kordas RL, Crim RN, Singh GG (2010). Meta-analysis reveals negative yet variable effects of ocean acidification on marine organisms. Ecology Letters.

[ref-38] Kroeker KJ, Kordas RL, Crim R, Singh GG (2013). Impacts of ocean acidification on marine organisms:quantifying sensitivities and interaction with warming. Global Change Biology.

[ref-39] Kvitt H, Kramarsky-Winter E, Maor-Landaw K, Zandbank K, Kushmaro A, Rosenfeld H, Tchernov D (2015). Breakdown of coral colonial form under reduced pH conditions is initiated in polyps and mediated through apoptosis. Proceedings of the National Academy of Sciences of the United States of America.

[ref-40] Law CS, Bell JJ, Bostock HC, Cornwall CE, Cummings VJ, Currie K, Davy SK, Gammon M, Hepburn CD, Catriona LH, Lamare M, Mikaloff-Fletcher SE, Nelson WA, Parsons DM, Ragg NLC, Sewell MA, Smith AM, Tracey DM (2018). Ocean acidification in New Zealand waters: trends and impacts. Journal of Marine and Freshwater Research.

[ref-41] Maier C, Bils F, Weinbauer MG, Watremez P, Peck MA, Gattuso JP (2013a). Respiration of Mediterranean cold-water corals is not affected by ocean acidification as projected for the end of the century. Biogeosciences.

[ref-42] Maier C, Hegeman J, Weinbauer MG, Gattuso JP (2009). Calcification of the cold-water coral Lophelia pertusa, under ambient and reduced pH. Biogeosciences.

[ref-43] Maier C, Popp P, Sollfrank N, Weinbauer MG, Wild C, Gattuso JP (2016). Effects of elevated pCO2 and feeding on net calcification and energy budget of the Mediterranean cold-water coral Madrepora oculata. Journal of Experimental Biology.

[ref-44] Maier C, Schubert A, Berzunza-Sànchez MM, Weinbauer MG, Watremez P, Gattuso J-P (2013b). End of the century pCO2 levels do not impact calcification in Mediterranean cold-water corals. PLOS ONE.

[ref-45] Maier C, Watremez P, Taviani M, Weinbauer MG, Gattuso JP (2012). Calcification rates and the effect of ocean acidification on Mediterranean cold-water corals. Proceedings of the Royal Society B.

[ref-46] Maier C, Weinbauer MG, Gattuso JP, Orejas C, Jiménez C Fate of Mediterranean cold-water corals as a result of global climate change. A synthesis. Mediterranean cold-water corals: past, present and future.

[ref-47] Marubini F, Ferrier-Pages C, Furla P, Allemand D (2008). Coral calcification responds to seawater acidification: a working hypothesis towards a physiological mechanism. Coral Reefs.

[ref-48] Mehrbach C, Culberson CH, Hawley JE, Pytkowicx RM (1973). Measurement of the apparent dissociation constants of carbonic acid in seawater at atmospheric pressure. Limnology and Oceanography.

[ref-49] Mikaloff-Fletcher SE, Bostock HC, Williams M, Forcen A (2017). Modelling the effects of ocean acidification in New Zealand. New Zealand aquatic environment and biodiversity report.

[ref-50] Milligan RJ, Spence GJ, Roberts JM, Bailey DM (2016). Fish communities associated with cold-water corals vary with depth and substratum type. Deep Sea Research Part I.

[ref-51] Movilla J, Orejas C, Calvo E, Gori A, López-Sanz À, Grinyó J, Domínguez-Carrió C, Pelejero C (2014). Differential response of two Mediterranean cold-water coral species to ocean acidification. Coral Reefs.

[ref-52] Orr JC, Fabry VJ, Aumont O, Bopp L, Doney SC, Feely RA, Yool A (2005). Anthropogenic ocean acidification over the twenty-first century and its impact on calcifying organisms. Nature.

[ref-53] Parker SJ, Bowden DA (2010). Identifying taxonomic groups vulnerable to bottom longline fishing gear in the Ross Sea Region. CCAMLR Science.

[ref-54] Purser A, Orejas C, Gori A, Tong R, Unnithan V, Thomsen L (2013). Local variation in the distribution of benthic megafauna species associated with cold-water coral reefs on the Norwegian margin. Continental Shelf Research.

[ref-55] Richardson AD, Braswell BH, Hollinger DY, Jenkins JP, Ollinger SV (2009). Near surface remote sensing of spatial and temporal variation in canopy phenology. Ecological Applications.

[ref-56] Roberts JM, Wheeler AJ, Freiwald A (2006). Reefs of the deep: the biology and geology of cold-water coral ecosystems. Science.

[ref-57] Schneider CA, Rasband WS, Eliceiri KW (2012). NIH Image to ImageJ: 25 years of image analysis. Nature Methods.

[ref-58] Soffker M, Sloman KA, Hall-Spencer JM (2011). *In situ* observations of fish associated with coral reefs off Ireland. Deep Sea Research I.

[ref-59] Sweetman AK, Thurber AR, Smith CR, Levin LA, Mora C, Wei CL, Gooday AJ, Jones DOB, Rex M, Yasuhara M, Ingels J, Ruhl HA, Frieder CA, Danovaro R, Würzberg L, Baco A, Grupe BM, Pasulka A, Meyer KS, Dunlop KM, Henry L-A, Roberts JM (2017). Major impacts of climate change on deep-sea benthic ecosystems. Elementa: Science of the Anthropocene.

[ref-60] Thresher RE, Tilbrook BD, Fallon S, Wilson NC, Adkins J (2011). Effects of chronic low carbonate saturation levels on the distribution, growth and skeletal chemistry of deep-sea corals and other seamount megabenthos. Marine Ecology Progress Series.

[ref-61] Tracey D, Bostock H, Currie K, Mikaloff-Fletcher S, Williams M, Hadfield M, Neil H, Guy C, Cummings V (2013). The potential impact of ocean acidification on deep-sea corals and fisheries habitat in New Zealand waters. New Zealand aquatic environment and biodiversity Report No. 117.

[ref-62] Tracey DM, Rowden AA, Mackay KA, Compton T (2011). Habitat-forming cold-water corals show affinity for seamounts in the New Zealand region. Marine Ecology Progress Series.

[ref-63] Turley CM, Roberts JM, Guinotte JM (2007). Corals in deep-water: will the unseen hand of ocean acidification destroy cold-water ecosystems?. Coral Reefs.

[ref-64] Williams A, Schlacher TA, Rowden AA, Althaus F, Clark MR, Bowden DA, Stewart R, Bax NJ, Consalvey M, Kloser RJ (2010). Seamount megabenthic assemblages fail to recover from trawling impacts. Marine Ecology.

[ref-65] Winters G, Holzman R, Blekhman A, Beer S, Loya Y (2009). Photographic assessment of coral chlorophyll contents: implications for ecophysiological studies and coral monitoring. Journal of Experimental Marine Biology and Ecology.

[ref-66] Wolankski E, Hamner WM (1998). Topographically controlled forces in the ocean and their biological influence. Science.

[ref-67] Wood R (1999). Reef evolution.

