# Supplementary material for: The physiological response of the deep-sea coral Solenosmilia variabilis to ocean acidification
Source: PeerJ. 2018 Jul 20;6:e5236. doi: 10.7717/peerj.5236 (PMC6055589; doi:10.7717/peerj.5236)
Supplement: Supplemental Information 1 — Pilot study and method development to measure the loss of coenenchyme. [file peerj-06-5236-s002.docx]

**Supplementary information**:

A pilot study was conducted where coral colonies from the treatment group (n = 4), with a visible loss in coenenchyme saturation over time, were profiled for colour intensity. Intensity of the green channel remained consistent over time, while intensity of the red channel decreased and intensity of the blue channel increased (Fig. S1).

To confirm that the visual loss in health corresponded to changes in the relative intensity of the blue and red colour channels, photos taken of the same four corals were then visually categorized on a scale from one to five. Corals in Category Four were the most healthy, showing pink or brown pigmentation on the skeleton and bright polyps. Corals in Category One were dead, with no pink or brown pigmentation. The percentage loss for each of these channels was then plotted against the health index assigned to that coral (Fig. S2). A decrease in relative intensity of the red channel corresponded to a reduction in coral health, whilst an increase in the relative intensity of the blue channel corresponded to a reduction in coral health. There appeared to be no relationship with the green colour channel.

Once is it was confirmed that a loss in intensity of the red colour channel corresponded to a loss in coral health (Fig. S1-S2), images were analysed for percentage change in relative intensity of the red colour channel using the following equation:

$$R_{J}=100 x \frac{{(S}_{J1}-S_{J2})}{\left( S_{J1} x \frac{T_{1}}{T_{2}} \right)}$$

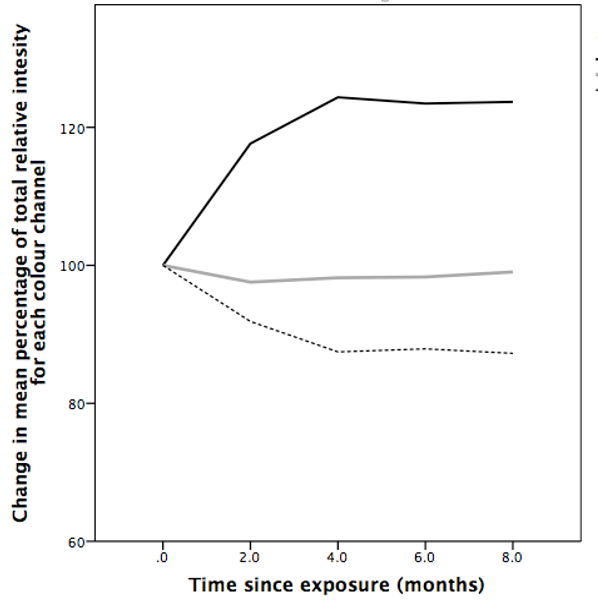
Where R_J_ = the relative intensity of the J^th^ colour channel (red, green or blue), S_J1_ = the mean intensity of the J^th^ colour channel at time-point one, S_J2_ = the mean intensity of the J^th^ colour intensity at time-point two, T_1_ = time-point one and T_2_ = time-point.

Figure S1: The relative change in intensity of each of the red, green and blue colour channels in images of *Solenosmilia variabilis* colony fragments, taken over 8 months. A pilot study (n=4) was conducted to determine how the relative intensity of each of the Red (broken line), Green (grey line) and Blue (black line) colour channels changed over time.


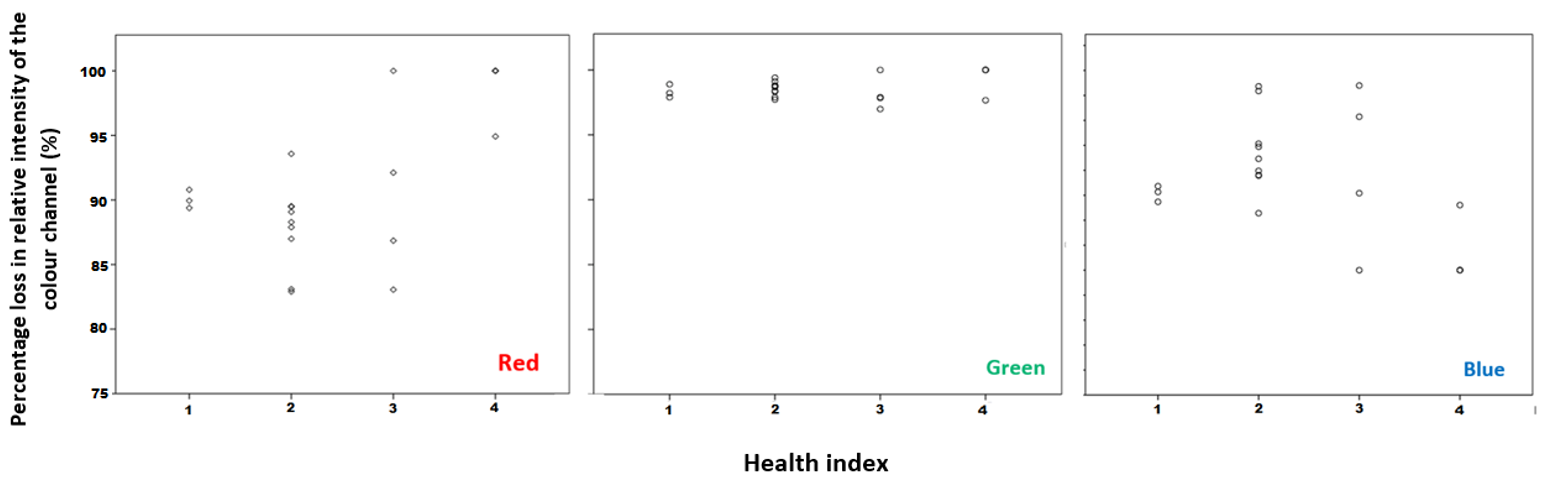
**Figure S2:** The relationship between coral health index of *Solenosmilia variabilis* fragments and the relative loss in intensity of each of the red, blue and green colour channels. These plots were used to confirm a loss in intensity of the red colour channel corresponded to a visual loss in coral health and coenenchyme
